# Current status and time trends of lipid and use of statins among older adults in China—real world data from primary community health institutions

**DOI:** 10.3389/fpubh.2023.1138411

**Published:** 2023-04-26

**Authors:** Junrong Jiang, Jun Huang, Hai Deng, Hongtao Liao, Xianhong Fang, Xianzhang Zhan, Shulin Wu, Yumei Xue

**Affiliations:** ^1^Guangdong Cardiovascular Institute, Guangdong Provincial People’s Hospital, Guangdong Academy of Medical Sciences, Guangzhou, China; ^2^Guangdong Provincial Key Laboratory of Clinical Pharmacology, Guangdong Provincial People’s Hospital, Guangdong Academy of Medical Sciences, Guangzhou, China; ^3^Department of Geriatrics, Guangdong Provincial People’s Hospital, Institute of Geriatrics, Guangdong Academy of Medical Sciences, Guangzhou, China

**Keywords:** lipid, statins, older adults, current status, China

## Abstract

**Background:**

Elevated serum total cholesterol and low-density lipoprotein cholesterol (LDL-C) levels are established risk factors for cardiovascular diseases, a leading cause of death in China, especially in aged population. We sought to assess the latest levels of serum lipids, prevalence of dyslipidemia and achievement of LDL-C lowering targets among Chinese aged population.

**Methods:**

The data was obtained from the annual health check and medical records in primary community health institutions of Yuexiu District, Guangzhou, Southern China. A sample of approximately 135,000 participants provides comprehensive estimates of the status of cholesterol level and statins use in older adults in China. Clinical characteristics were compared by different age grades, genders and years. Independent risk factors associated with statin use were determined by stepwise logistic regression analysis.

**Results:**

The mean levels of TC, HDL-C, LDL-C, TG were 5.39, 1.45, 3.10, and 1.60 mmol/L, respectively, while the prevalence of high TC, high TG, high LDL-C, and low HDL-C were 21.99, 15.52, 13.26, and 11.92%, respectively. Although statin use showed an increasing trend in both participants > 75 years and ≤75 years of age, the achievement of treatment goals fluctuated between 40.94 and 48.47%, and even seemed to have a downward trend. Stepwise multiple logistic regression analysis further indicated that age, medical insurance, ability of self-care, hypertension, stroke, CAD, and high LDL-C were shown to be associated with statins use (*P* < 0.05). Those aged ≤75 years old seemed to be less likely to use statin, and those without medical insurance or ability of self-care seemed to be less likely to use statin, too. Patients with hypertension, stroke, CAD and high LDL-C were more inclined to use statins.

**Conclusion:**

Chinese aged population currently experienced high serum lipid levels and prevalence of dyslipidemia. Although an increasing trend was shown in the proportion of high CVD risk and statin use, the achievement of treatment goals seemed to have a downward trend. Improvement of lipid management is necessary in order to reduce the burden of ASCVD in China.

## 1. Introduction

Cardiovascular disease (CVD), of which Atherosclerotic CVD (ASCVD) is the major component, has rapidly and substantially increased in China. There were approximately 2.4 million deaths from ASCVD in 2016, more than 1-fold increase from 1990, accounting for 61% of CVD deaths and 25% of all deaths (1). Over the past decades, the health status of general population in China has significantly improved, with substantial increases in life expectancy and healthy life expectancy (2). This improvement has led to a rapid and consistent increase in the aging population, and thus has increased the burden of CVD. Thanks to the population growth and aging, the projected annual CVD events in China may increase by >50% between 2010 and 2030 (3).

Since serum cholesterol was identified as one of the “risk factors” for coronary heart disease in 1961 (4), many epidemiological studies and randomized clinical trials have further confirmed that elevated low-density lipoprotein cholesterol (LDL-C) is the main risk factor of ASCVD (5, 6). A meta-analysis of observational studies showed that higher cholesterol level was associated with increased CVD mortality at all ages, while statin therapy produces significant reductions in major vascular events irrespective of age (7).

Since China has the largest aged population in the world, in order to reduce the risk of CVD in the population, the status of cholesterol levels and statin use are noteworthy. To this end, we sought to estimate the status of cholesterol levels as well as statin use among older adults in China by analyzing data from the annual health check and medical records in primary community health institutions.

## 2. Methods

### 2.1. Data source and study population

With the authorization of the local government and Center for Disease Control and Prevention (CDC), the data was obtained from the annual health check and medical records in primary community health institutions of Yuexiu District, Guangzhou, Southern China. The study was approved by the ethics committee of Guangdong Provincial People’s Hospital and was conducted in accordance with the Declaration of Helsinki and Good Clinical Practice Guidelines. The requirement for informed consent from patients whose information was retrospectively collected was waived.

The annual health check, funded by the local government, aims to assess the prevalence and changes over time of major non-communicable and chronic diseases (NCDs) and their risk factors in the older adults. Participation is voluntary for those over 65 years old. Laboratory tests (blood routine, liver and kidney function, electrolytes, lipids, etc.), ECG and chest radiograph are included in the annual health check. The annual health check was serviced throughout the year and all costs were covered by the government. Participants got their report from the community health institutions, the community physicians explained the results to them and provided medical advice. Based on available data, participants did not appear to have decreased during the COVID-19 pandemic.

**Figure 1:**
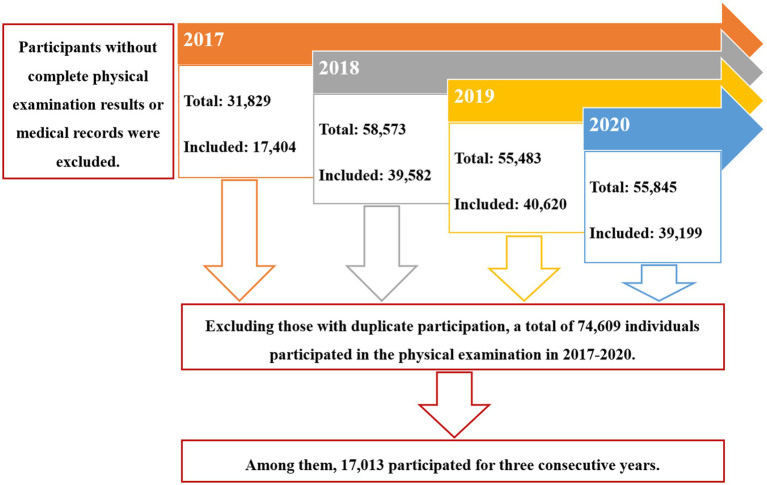
Flow diagram of participants.

The resident population in Yuexiu district is about 1.15 million, with 13% of the population over 65 years old. There were 18 primary community health institutions involving in this study. Data from 2017–2020 were included. In 2017–2020, 31,829, 58,573, 55,483, and 54,845 participants underwent the annual health check, respectively. Participants without complete annual health check results or medical records in primary community health institutions were excluded. Consequently, 17,404, 39,582, 40,620, and 39,199 participants were included from 2017 to 2020. Excluding those with duplicate participation, a total of 74,609 individuals participated in the annual health check in 2017–2020. Among them, 17,013 participated in the annual health check for three consecutive years (Figure 1).

### 2.2. Data collection

Information on sociodemographic characteristics (age, sex, ethnicity, body mass index, level of education, marital status, medical insurance coverage), medical history, including hypertension, diabetes mellitus (DM), established coronary artery disease (CAD), previous stroke/transient ischemic attack (TIA), ability of self-care, history of smoking, and alcohol consumption, exercise, as well as medicine use, was collected for each participant from medical records and annual health check each year. Data collections of serum cholesterol levels and use of statin were based on the results of the annual health check. Direct measurement LDL-C was used in this study.

The serum total cholesterol (TC), LDL-C, high-density lipoprotein cholesterol (HDL-C), and triglyceride (TG) levels were classified according to the Chinese Guideline for the Management of Dyslipidemia in Adults (8). High TC was defined as TC ≥ 6.22 mmol/L. High LDL-C was defined as LDL-C ≥ 4.14 mmol/L. Low HDL-C was defined as LDL-C < 1.04 mmol/L and high TG was defined as TG ≥ 2.26 mmol/L. The CVD risk stratification and the therapeutic targets were also identified by the recommendations of the Chinese guideline (8, 9). Patients with a diagnosis of ASCVD were considered to have a very high risk, whereas patients with LDL-C ≥ 4.9 mmol/L or TC ≥ 7.2 mmol/L were classified as high-risk. For patients with diabetes mellitus, patients aged ≥40 years with LDL-C ≥ 1.8 mmol/L or TC ≥ 3.1 mmol/L were directly classified as high-risk. Others were assessed according to age, gender, history of hypertension, smoking status, HDL-C, TC, and LDL-C levels. Individuals were classified into high-risk group (10-year risk for ASCVD ≥10%), moderate risk group (10-year risk for ASCVD 5–9%), and low-risk group (10 year risk for ASCVD < 5%). The therapeutic targets for LDL-C were < 1.8 mmol/L for very high risk, <2.6 mmol/L for high-risk, and <3.4 mmol/L for low and moderate risk.

### 2.3. Statistical analysis

Clinical characteristics were reported as mean ± SD for continuous variables and proportions for categorical variables and compared by different age grades, genders and years. Continuous variables were compared using independent *t* test, and categorical variables were compared using χ^2^ test and log-rank test. Independent risk factors associated with statin use were determined by stepwise logistic regression analysis. The odds ratios and 95% confidence intervals were estimated to assess the magnitude of the association between these factors and the use of statins. Data analysis was performed using SAS 9.4. Statistical significances for differences were defined as a two-sided *p* value of less than 0.05. Measures of performance were summarized using two-sided 95% confidence intervals.

## 3. Results

### 3.1. Baseline characteristics and overview lipid status

Baseline characteristics of participants in 2017–2020 are shown in Table 1. A total of 74,609 participants (40.98% men and 59.02% women; 67.57% ≤75 years old and 32.43% >75 years old) were included in the analysis. The mean levels of TC, HDL-C, LDL-C, TG were 5.39, 1.45, 3.10, and 1.60 mmol/L, respectively, while the prevalence of high TC, high TG, high LDL-C and low HDL-C were 21.99, 15.52, 13.26, and 11.92%, respectively. A significant difference was observed between men and women for all characteristics (*p* < 0.05). More men were educated and married, and more men took regular exercises. Hypertension, diabetes and CAD were more common in women, while men had a higher proportion of overweight and stroke. Although women had higher lipid levels (TC, TG, LDL-C, and HDL-C), more men were at high or very high risk of CVD. More men used statin and achieved LDL-C lowering target.

**Table 1:** Baseline characteristics of participants by age and sex in 2017–2020.

	Total	Age		Sex		*p* value for age	*p* value for sex
		≤75 years	>75 years	Male	Female		
*N* (%)	74,609	50,410 (67.57%)	24,199 (32.43%)	30,575 (40.98%)	44,034 (59.02%)	–	–
Male (%)	30,575 (40.98%)	21,198 (42.06%)	9,377 (38.75%)	–	–	–	–
Age > 75 years (%)	24,199 (32.43%)	–	–	9,377 (30.67%)	14,822 (33.66%)	–	–
Education (high school or above) (%)	26,384 (35.36%)	19,485 (38.65%)	6,899 (28.51%)	13,492 (44.13%)	12,892 (29.28%)	<0.01	<0.01
Married (%)	61,366 (82.25%)	44,489 (88.25%)	16,877 (69.74%)	28,219 (92.30%)	33,147 (75.28%)	<0.01	<0.01
Exercise (%)	44,200 (59.24%)	31,806 (63.09%)	12,394 (51.22%)	18,823 (61.56%)	25,377 (57.63%)	<0.01	<0.01
Overweight (%)	32,823 (43.99%)	23,571 (46.76%)	9,252 (38.23%)	13,910 (45.49%)	18,913 (42.95%)	<0.01	<0.01
Hypertension (%)	40,847 (54.75%)	25,439 (50.46%)	15,408 (63.67%)	16,054 (52.51%)	24,793 (56.30%)	<0.01	<0.01
Diabetes (%)	15,671 (21.00%)	10,031 (19.90%)	5,640 (23.31%)	6,294 (20.59%)	9,377 (21.29%)	<0.01	0.02
CAD (%)	11,779 (15.79%)	6,705 (13.30%)	5,074 (20.97%)	4,632 (15.15%)	7,147 (16.23%)	<0.01	<0.01
Stroke (%)	1,964 (2.63%)	1,007 (2.00%)	957 (3.95%)	980 (3.21%)	984 (2.23%)	<0.01	<0.01
Current smokers (%)	5,336 (7.15%)	4,447 (8.82%)	889 (3.67%)	5,173 (16.92%)	163 (0.38%)	<0.01	<0.01
Drinking (%)	5,276 (7.07%)	4,230 (8.39%)	1,046 (4.32%)	4,691 (15.34%)	585 (1.33%)	<0.01	<0.01
TC (mmol/L)	5.39 ± 1.16	5.49 ± 1.15	5.20 ± 1.17	5.13 ± 1.10	5.58 ± 1.17	<0.01	<0.01
TG (mmol/L)	1.60 ± 1.13	1.66 ± 1.21	1.48 ± 0.94	1.55 ± 1.18	1.64 ± 1.10	<0.01	<0.01
LDL-C (mmol/L)	3.10 ± 0.95	3.18 ± 0.94	2.93 ± 0.94	2.98 ± 0.91	3.18 ± 0.97	<0.01	<0.01
HDL-C (mmol/L)	1.45 ± 0.39	1.45 ± 0.39	1.45 ± 0.41	1.35 ± 0.37	1.52 ± 0.40	0.42	<0.01
High TC (%)	16,410 (21.99%)	12,101 (24.01%)	4,309 (17.81%)	4,464 (14.60%)	11,946 (27.13%)	<0.01	<0.01
High TG (%)	11,582 (15.52%)	8,614 (17.09%)	2,968 (12.26%)	4,287 (14.02%)	7,295 (16.57%)	<0.01	<0.01
High LDL-C (%)	9,894 (13.26%)	7,465 (14.81%)	2,429 (10.04%)	3,084 (10.09%)	6,810 (15.47%)	<0.01	<0.01
Low HDL-C (%)	8,896 (11.92%)	5,705 (11.32%)	3,191 (13.19%)	5,442 (17.80%)	3,454 (7.84%)	<0.01	<0.01
Risk stratification						<0.01	0.02
Low or moderate	42,904 (57.51%)	30,015 (59.54%)	12,889 (53.26%)	17,428 (57.00%)	25,476 (57.86%)		
High or very high	31,705 (42.49%)	20,395 (40.46%)	11,310 (46.74%)	13,147 (43.00%)	18,558 (42.14%)		
Statin use (%)	3,362 (4.51%)	2,227 (4.42%)	1,135 (4.69%)	1,449 (4.74%)	1,913 (4.34%)	0.09	0.01
Achievement of treatment goals (%)	34,456 (46.18%)	22,626 (44.88%)	11,830 (48.89%)	15,190 (49.68%)	19,266 (43.75%)	<0.01	<0.01

Similarly, except for statin use, there were significant differences in all characteristics between participants >75 and ≤75 years of age. More participants ≤75 years of age were educated and married, and more participants ≤75 years of age took regular exercises. Hypertension, diabetes, CAD as well as stroke were more common in participants >75 years of age, while participants ≤75 years of age had higher lipid levels (TC, TG, and LDL-C). Although more participants >75 years of age were at high or very high risk of CVD, the proportion of achieving LDL-lowering target was higher.

### 3.2. Annual analysis, trends on lipids level and statins therapy

**Figure 2:**
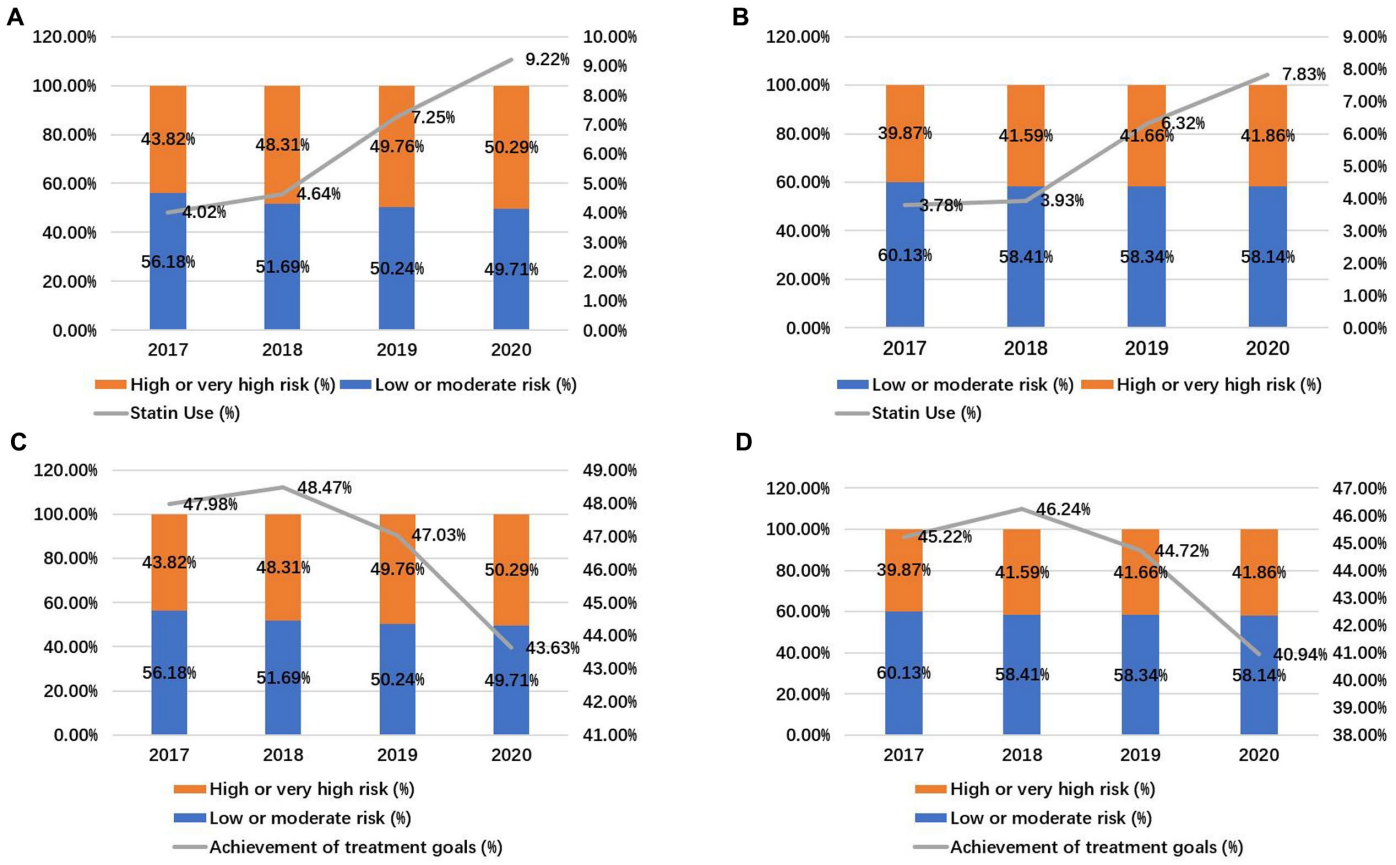
Statin use and achievement of treatment goals in older adults. Statin use in participants > 75 years old **(A)** and ≤75 years old **(B)**. Achievement of treatment goals in participants > 75 years old **(C)** and ≤75 years old **(D)**.

Cholesterol levels in older adults of each year are shown in Table 2. The proportion of people ≤75 years seemed to increase year by year (*p* < 0.01), while there was no significant difference in the gender composition in different years. There were significant differences in cholesterol levels, statin use as well as achievement of treatment goals among different years (*p* < 0.05). Among these, the proportions of high-risk and very high-risk groups have increased year by year. Although statin use showed an increasing trend in both participants >75 years and ≤75 years of age (Figures 2A,B), the achievement of treatment goals fluctuated between 40.94 and 48.47%, and even seemed to have a downward trend (Figures 2C,D).

**Table 2:** Cholesterol levels in older adults of each year.

		2017 (*N* = 17,404)	2018 (*N* = 39,582)	2019 (*N* = 40,620)	2020 (*N* = 39,199)	*p* value
Age grade (%)	≤75 years	10,983 (63.11%)	25,490 (64.40%)	27,164 (66.87%)	26,655 (68.00%)	<0.01
	>75 years	6,421 (36.89%)	14,092 (35.60%)	13,456 (33.13%)	12,544 (32.00%)	
Gender (%)	Male	6,968 (40.04%)	16,038 (40.52%)	16,333 (40.21%)	15,791 (40.28%)	0.70
	Female	10,436 (59.96%)	23,544 (59.48%)	24,287 (59.79%)	23,408 (59.72%)	
TC (mmol/L)		5.41 ± 1.13	5.41 ± 1.16	5.34 ± 1.15	5.39 ± 1.18	<0.01
LDL-C (mmol/L)		3.08 ± 0.91	3.06 ± 0.93	3.09 ± 0.95	3.20 ± 0.99	<0.01
HDL-C (mmol/L)		1.49 ± 0.41	1.44 ± 0.38	1.44 ± 0.38	1.45 ± 0.39	<0.01
High TC (%)		3,637 (20.90%)	8,801 (22.23%)	8,625 (21.23%)	9,165 (23.38%)	<0.01
High LDL-C (%)		2,079 (11.95%)	4,470 (12.05%)	5,370 (13.22%)	6,713 (17.13%)	<0.01
Low HDL-C (%)		1,745 (10.03%)	4,906 (12.39%)	4,985 (12.27%)	4,630 (11.81%)	<0.01
Risk stratification (%)	Low or moderate	10,211 (58.67%)	22,173 (56.02%)	22,608 (55.66%)	21,734 (55.45%)	<0.01
	High or very high	7,193 (41.33%)	17,409 (43.98%)	18,012 (44.34%)	17,465 (44.55%)	
Statin use (%)		673 (3.87%)	1,656 (4.18%)	2,693 (6.63%)	3,243 (8.27%)	<0.01
Achievement of treatment goals (%)		8,047 (46.24%)	18,616 (47.03%)	18,478 (45.49%)	16,386 (41.80%)	<0.01
Achievement of treatment goals (%) (in high or very high risk group)		1,390 (19.32%)	3,815 (21.91%)	3,659 (20.31%)	3,286 (18.81%)	<0.01

### 3.3. Clinical factors associated with statins use in older adults

**Figure 3:**
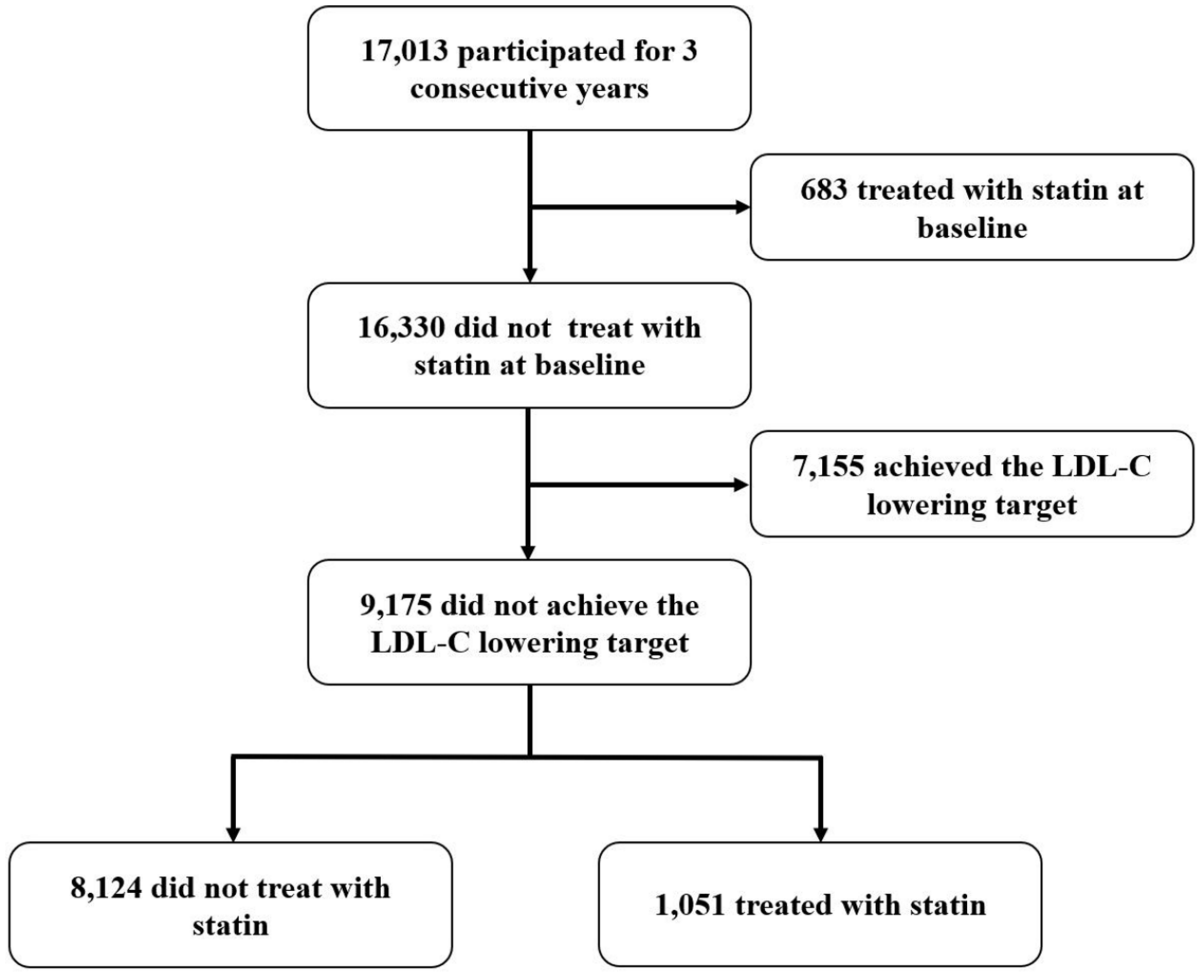
Overview of the participants with three consecutive years observation.

In this study, 17,013 participated in the annual health check for three consecutive years (Figure 3). 683 treated with statin and 7,155 achieved the LDL-C lowering target at baseline. For those without statin therapy at baseline, 1,051 started statin therapy during the study period, while 8,124 did not receive statin treatment all the time. Stepwise multiple logistic regression analysis further indicated that age, medical insurance, ability of self-care, hypertension, stroke, CAD and high LDL-C were shown to be associated with statins use (*p* < 0.05) (Table 3). Those aged ≤75 years old seemed to be less likely to use statin, and those without medical insurance or ability of self-care seemed to be less likely to use statin, too. Patients with hypertension, stroke, CAD, and high LDL-C were more inclined to use statins. The results of the stepwise multiple logistic regression are shown in Table 3.

**Table 3:** Clinical factors associated with statins use in older adults.

		ALL (*N* = 9,175)	Statins (*N* = 1,051)	No statins (*N* = 8,124)	*p* value	Odds ratio (no statins)	95% CI
Age grades	≤75	6,515	730	5,785	0.02	1.19 (≤75 vs. >75)	1.03–1.38
	>75	2,660	321	2,339			
Gender	Male	3,299	381	2,918	0.65	–	–
	Female	5,876	670	5,206			
Overweight	Yes	4,303	525	3,778	0.26	–	–
	No	4,872	526	4,346			
Education	Below high school	6,248	697	5,551	0.40	–	—
	Above high school	2,927	354	2,573			
Marital status	Married	7,273	847	6,426	0.08	–	–
	Unmarried	1,902	204	1,698			
Medical insurance	Yes	8,735	1,035	7,700	0.02	1.87 (no vs. yes)	1.12–3.13
	No	440	16	424			
Ability of self-care	Yes	8,968	1,033	7,935	0.02	1.81 (no vs. yes)	1.09–3.01
	No	207	18	189			
Exercise	No or rarely	3,836	452	3,834	0.43	–	–
	Regularly	5,339	599	4,740			
Smoking status	No or quitted	8,678	1,006	7,672	0.11	–	–
	Current smokers	497	45	452			
Alcohol	Yes	562	57	505	0.63	–	–
	No	8,613	994	7,619			
Hypertension	Yes	5,773	893	4,880	<0.01	0.34 (yes vs. no)	0.28–0.40
	No	3,402	158	3,244			
Diabetes	Yes	2,776	326	2,450	0.10	–	–
	No	6,399	725	5,674			
Stroke	Yes	403	93	310	<0.01	0.40 (yes vs. no)	0.31–0.52
	No	8,772	958	7,814			
CAD	Yes	2,397	550	1,847	<0.01	0.30 (yes vs. no)	0.26–0.35
	No	6,778	501	6,277			
High TC	Yes	3,280	352	2,928	0.08	–	–
	No	5,895	699	5,196			
High LDL-C	Yes	1,989	245	1,744	<0.01	0.52 (yes vs. no)	0.43–0.64
	No	7,186	806	6,380			
Multiple drugs	Yes	3,602	546	3,056	0.29	–	–
	No	5,573	505	5,068			

## 4. Discussion

This study with a sample of 74,609 participants and approximately 135,000 health checks provided comprehensive estimates of the status of cholesterol level and statins use in older adults in China. The mean levels of TC, HDL-C, LDL-C, TG were 5.39, 1.45, 3.10, and 1.60 mmol/L, respectively. These levels were much higher than those reported by previous studies conducted in the Chinese population from 1983 to 2014 (10–12). A deteriorating trend was observed in the past decades because of the lifestyle changes caused by the rapid economic growth and industrialization. The lipids levels in this study were comparable to those reported in the United States (TC and LDL-C were 5.08 and 3.00 mmol/L, respectively) (13) and Japan (TC and LDL-C were 5.18 and 2.99 mmol/L, respectively) (14).

Higher lipid levels (TC, TG, LDL-C, and HDL-C) were observed in people ≤75 years and women. However, more people >75 years and men were at high or very high risk of CVD, with higher proportion of achieving LDL-lowering target. Menopause has been reported to lead to changes in lipid profile through reducing HDL-C, and elevating TC, TG and LDL-C (15). The higher cholesterol level in female may be partly explained by the difference of life style (less exercise in female), eating habit, concomitant metabolic syndrome and possibility of difference in statin response among gender in the elderly. Besides, more effective lipid-lowering treatment might be used in patients at high or very high risk of CVD.

In this study, an increasing trend was shown in the proportion of high CVD risk and statin use (*p* < 0.01, from 41.33 to 44.55% for high CVD risk and from 3.80 to 8.45% for statin use), while the achievement of treatment goals fluctuated between 40.94 and 48.47%, and even seemed to have a downward trend. As the use rate of statin increased, the rate of reaching target LDL-C goal did not increase. Among these population, about 20% with high CVD risk or very high CVD risk did not achieve the LDL-lowering goals in 2017–2020. The achievements of LDL-lowering targets from different studies were different (16, 17). The achievements of LDL-lowering targets from general population living in communities seemed to be lower. A previous study from communities in China showed that 74.5% with high CVD risk and 93.2% with very high CVD risk did not achieve the LDL-lowering goals, consistent with this study (12). Similar to anticoagulation rates in atrial fibrillation (18), statin use has also shown an upward trend in the dyslipidemic population. However, considering failure to attain their LDL-C goals in a large population, the statin use was still quite low. One explanation for this result may be the physicians’ poor understanding of the guideline recommended LDL-C goal (19). Since the achievements of LDL-lowering targets did not increase with the increase of statin use, statin use alone may not be sufficient for the aged population.

Age, medical insurance, ability of self-care, hypertension, stroke, CAD and high LDL-C were shown to be independently associated with statins use in older adults in this study. Those aged ≤75 years old seemed to be less likely to use statin, and those without medical insurance or ability of self-care seemed to be less likely to use statin, too. Patients with hypertension, stroke, CAD and high LDL-C were more inclined to use statins. This suggested that more patients used statins for secondary prevention. Recent evidence has strengthened the role of LDL-C as a risk factor for ASCVD in older adults (20). Despite the evidence that statins significantly reduce major vascular events regardless of age, there is still less direct evidence of statin benefit in patients without evidence of ASCVD (7, 21). For older adults at high risk for cardiovascular disease, primary prevention with statins is a reasonable option after considering other factors such as risk modifiers, frailty, estimated lifetime benefits, comorbidities, and patient preferences (22). Notably, although the traditional LDL-C target of <2.6 mmol/L (100 mg/dL) seems reasonable, there is insufficient evidence to support the goal of primary prevention in older patients in terms of LDL-C targets (22). More related information will be provided by ongoing randomized and controlled trials (STAREE and PREVENTABLE) (23).

## 5. Limitation

This study has several limitations. First, since the data was derived from Guangzhou, one of the economic centers of China, the results only reflected the lipid level and statin use of a relatively developed area in China and may not fully be representative of the whole country, especially less-developed regions. Besides, by the reasons that participants who came to perform health screening might have more health awareness, be more transportable or be more likelihood to receive a notification of health screening. This would make the prevalence of dyslipidemia overestimated. Based on the limited available data, this study demonstrated higher lipid levels and similar LDL-C control rates compared to previous studies from China (10–12). Second, the effect of non-statin lipid lowering agents on the lipid profile as well as the influence of attending physicians on statin prescription were not analyzed in this study. Third, the secondary causes of dyslipidemia were not explored, therefore, these might be included into the study population. Finally, due to data limitations, this study was not able to analyze the relationship between risk factors and dyslipidemia development. In addition, statins selection for initiation or changing overtime in the elderly are needed to be further explored.

## 6. Conclusion

In conclusion, this study showed the high serum lipid level and prevalence of dyslipidemia in older adults in China currently. Although an increasing trend was showed in the proportion of high CVD risk and statin use, the achievement of treatment goals seemed to have a downward trend. Age, medical insurance, ability of self-care, hypertension, stroke, CAD, and high LDL-C were shown to be independently associated with statins use in older adults in this study. Improvement of lipid management is necessary in order to reduce the burden of ASCVD in China.

## Data availability statement

The original contributions presented in the study are included in the article/supplementary material, further inquiries can be directed to the corresponding authors.

## Ethics statement

The studies involving human participants were reviewed and approved by the Ethics Committee of Guangdong Provincial People’s Hospital. Written informed consent for participation was not required for this study in accordance with the national legislation and the institutional requirements.

## Author contributions

JJ and JH collected the data and drafted the manuscript. YX and SW conceived the study. HL and XF analyzed the data. XZ and HD interpreted the results. All authors contributed to the article and approved the submitted version.

## Funding

This work was supported by the Science and Technology Programs of Guangdong Province (no. 2019B020230004), National Natural Science Foundation of China (no. 81870254), and the Zhongnanshan Medical Foundation of Guangdong Province (grant number no. 202151).

## Conflict of interest

The authors declare that the research was conducted in the absence of any commercial or financial relationships that could be construed as a potential conflict of interest.

## Publisher’s note

All claims expressed in this article are solely those of the authors and do not necessarily represent those of their affiliated organizations, or those of the publisher, the editors and the reviewers. Any product that may be evaluated in this article, or claim that may be made by its manufacturer, is not guaranteed or endorsed by the publisher.
